# Analysis of self-evaluated ethical competence of midwifery students at a selected nursing college in the Free State

**DOI:** 10.4102/curationis.v41i1.1925

**Published:** 2018-08-29

**Authors:** Moliehi R. Mpeli

**Affiliations:** 1 School of Nursing, Faculty of Health Sciences, University of the Free State, South Africa

## Abstract

**Background:**

It is imperative to know whether the students who have undergone a specific training perceive themselves as confident and competent in handling ethical dilemmas, in the face of contemporary ethical challenges. Such evaluation is significant especially for nursing and midwifery students who have undergone training that stipulates adherence to a code of ethics and professional norms. At present, such knowledge is limited, and this has an impact for ethics education.

**Objectives:**

The article aims to describe the self-evaluated ethical competence of midwifery students and to contrast the findings to the content of the ethics instruction received. Based on outcomes, the article aims to convey the claim to nursing institutions that current strategies that rely on teaching nursing ethics without appraising the context of a situation are ineffective in fostering ethical competence amongst students.

**Method:**

This study made use of a set of self-reflection reports in which the midwifery nursing students narrated their experiences in handling ethical issues.

**Results:**

Analysis of the self-reflective reports revealed that one of the three dimensions of ethical competence was limited. There was evidence of moral perception, moral action and substandard moral reasoning. The principles that were mostly referred to within the narratives were autonomy and beneficence.

**Conclusion:**

The findings support the argument that teaching principlism and enforcing a code of ethics without contextualising it coerces the student to conform without questioning their beliefs. Thus, ethical competence amongst the midwifery students may be described in terms of compliance to principles with limited reflection on the situation as a whole.

## Introduction

Self-evaluation regarding ethical competence and moral dispositions may be deemed subjective regarding the self; however, the activity is considered beneficial, because it is likely to reveal the real self and may influence the future self, as one is more likely to act in accordance with one’s own intentions (Armitage et al. 2008). In supporting the use of self-assessment in moral issues, Jordan, Leliveld and Tenbrunsel (2015:4) indicate that in this realm the ideal self is alleged to guide people’s behaviour and the nature of their self-assessments. Regardless of such a benefit, Grady et al. (2008), commenting from a US context, indicate that little is known about how nurses and social workers who have received a specific type of ethics training perceive their own ethical competence in dealing with ethical issues. Grady et al. (2008) posit that gathering such information may inform the precise effectiveness of ethics education, and therefore they devised resources for ethics support.

White, Phakoe and Rispel (2015) suggest that the majority of nurses are acquainted with the international codes of ethics for nurses and yet do not rely on them for their ethical practice. As Erasmus (2008), Mackenzie (2008) and Geyer (2015) highlight that the practice of nursing care (both in the public and in the private health system) in South Africa is dysfunctional, as it is charged with negligence and disgraceful conduct, and these authors lament for the face of nursing to change. As it is, it is difficult for ethics educators to determine whether students can apply the theoretical aspect of ethics to the real issues of midwifery practice. Indeed, such information is essential, because the midwifery practice exposes students to situations that call for training on ethical conduct. As the United Nations Educational, Scientific and Cultural Organization Asia and Pacific Regional Bureau for Education (2002) suggests, the education of nurses must not be aimed solely at academic achievement but must also develop students’ abilities to judge ethical issues.

In dealing with the challenges of ethical judgement, many authors are of the opinion that the traditional ethical principles and codes of ethics are ineffective in substantiating the moral decision-making of practising nurses and midwives in the face of contemporary nursing practices (Esterhuizen 1996; Goethals, Gastmans & Dierckx de Casterlé 2010; Pattison & Wainwright 2010). Inability of practising nurses and midwives to wield moral discernment and apply ethical principles and codes of ethics may be attributed to the way ethics is taught and assessed. Thus, there has been a call to engage students with new approaches that may facilitate this aspect of ethical competence. Ethical competence is understood as the ability to identify ethical issues, enact suitable actions and justify one’s assertions within a specific spatial and temporal context in which one is also able to recognise and reflect upon the feelings that ascertain what is good and bad in a situation (Jormsri et al. 2005; Kulju et al. 2016; Meine & Dunn 2013). Self-reflective ethical competence may be used as one of the resources of ethical development. Concurring with the use of self-reflective assessments, De las Fuentes, Willmuth and Yarrow (2005) confirm that the ability to explore one’s own moral and ethical values and attitudes, interpersonal skills of flexibility, openness to new ideas and change is a vital aspect of ethics education. In addition, it is believed that there is some sort of compensatory pattern of moral behaviour if one perceives that his or her moral self-worth is threatened or is too high (Sachdeva, Iliev & Medin 2009). Thus, it is essential to afford students the opportunity of evaluating their own ethical competence and to analyse such an assessment against the components of ethical competence.

Lechasseur et al. (2016) and Jormsri et al. (2005) indicate that ethical competence has three dimensions that are considered as key components. These dimensions are moral perception, moral judgment and moral behaviour, and they require the coordination of diverse skills, knowledge and attitudes for ethical decision-making (Jormsri et al. 2005). As De las Fuentes et al. (2005) attest, identification of these dimensions may enable evaluation and appraisal of ethical competency in a given situation. Thus, understanding the dimensions of ethical competence as core components may be used as criteria for evaluating ethical competence.

## Dimensions of ethical competence

The first dimension is moral perception, a dimension in which one is able to recognise a moral issue in a given situation (Jormsri et al. 2005). Moral perception is considered to be affective in nature, because it involves an experience of emotions in which one becomes aware of values and their expression in a given setting (Jormsri et al. 2005). In clarifying this dimension, Lechasseur et al. (2016) illustrate that the ability to recognise an ethical issue and its aspects in a situation is influenced by one’s ethical sensitivity, an aspect that is imbued with compassion. Ethical sensitivity relates to the ability to identify ethical issues and their consequences. Wisnewski (2015) attests that sympathy is also involved, or an emotional perception that enables one to perceive a moral issue.

The second dimension is called ‘moral judgement’. According to Campbell (2007) moral judgement is a process that unifies a multifaceted state of expressions involving moral beliefs, moral motivation, as well as emotions. It is within this process of moral judgement that the possible actions of an individual are evaluated as good or bad, with respect to the established norms and values or set of virtues informing a culture or subculture (Haidt 2001). Thus, an established ethical framework, whether deontology, principlism or a code of ethics, is essential for moral judgement. Saunders (2009) argues that the reflective equilibrium is a credible and acceptable normative model that is responsive to reason and may account for moral judgement in a given situation. Reflective equilibrium as a model for moral justification values and emphasises the use of all ethical principles and theories and diversified strategies for bringing about coherence and soundness to the decision-making, especially in situations of competing values and norms (Campbell 2014). As Campbell (2014) attests, judgments about a particular case remain stable and consistent only if the process of reflective equilibrium is achieved. This process is achieved through harmonising the total set of principles and the empirical claims involved.

The third dimension entails a moral action, which considers whether rational activities or endeavours are morally right or wrong and also judged to be morally good or bad by all stakeholders in a given situation (Spielthenner 2005). For example, within the context of midwifery, eliciting the type of behaviour conducive to resolving an identified ethical tension would constitute a moral act (Jormsri et al. 2005). Such an action would be based on deliberative reflection on acquired ethical knowledge and a variety of alternative solutions, as well as their implications in promoting the welfare of the individual (Lechasseur et al. 2016). These dimensions of ethical competency are complex and have presented many educators with a challenge concerning ethics education.

## Challenges for ethics education

In South Africa, the nursing qualification requirements for inclusion in the National Qualifications Framework are prescribed by the South African Nursing Council. According to the qualification framework, the exit level outcomes for the bachelor’s degree in nursing and midwifery stipulate that students should be able to identify and address ethical and legal issues based on critical reflection on ethical value (and legal) systems suitable to the nursing and midwifery practice within the legal framework of the country (SANC 2013). However, Iacobucci et al. (2012) are of the opinion that the implementation of the guidelines within the nursing qualification framework is subjective to faculty interpretation, resulting in ethics education that varies from one institution to the other.

The current practice of ethics education in one of the nursing education institutions in the Free State Province, based on the above guidelines, follows a curriculum consisting of eight credits or 80 notional hours of ethics teaching (South African Nursing Council 2013). The institution follows a theoretical approach to ethics education. The basis of the theoretical approach is the four ethical principles (autonomy, beneficence, non-maleficence and justice) comprising the ethical framework named ‘principlism’ as advocated by Beauchamp and Childress (2013), and this is in line with medical ethics, as defined by the goals of medicine. Furthermore, the students are introduced to and expected to subscribe to a code of ethics recently revised by the South African Nursing Council (2013). This code of ethics holds the professional to the roles of advocacy, accountability and consistent application of the standards of nursing and midwifery practice in the care for patients and their families (*Nursing Act* 33 of 2005). It is imperative to recognise that the codes of ethics are the codification of agreed principles that may be used to guide discussion and identification of right and wrong. As such, codes of ethics are never assumed to enact behavioural change, as needed for the developmental process of ethical competence. These theoretical ethical frameworks are always knowledge-based courses. Bertolami (2004) argues that knowledge-based courses accomplish little in ensuring exemplary conduct, because there is an enormous disconnect between knowing what is right and doing it – between understanding the principles of ethics at an intellectual level and applying them in daily life. Students are expected to internalise the ethical values and principles, which will enable them to demonstrate ethical competence during their interaction with patients. It is necessary to incorporate self-evaluation as one way of getting feedback, awareness and appreciation of one’s acquisition of ethical development, as proposed by De las Fuentes et al. (2005).

It is evident that there are challenges presented by the codes of ethics in substantiating moral decision-making. In addition, the codes of ethics do not encourage ethical reflection, as one has to conform, and as such it is problematic to solely rely on these knowledge-based courses for daily life situations. One aspect of concern is the complexity of ethical competence, which includes cognitive and affective skills, the aspects that make it difficult for educators to assess the extent to which students have acquired ethical competence. As it is, there are challenges surrounding the current ethical framework in developing ethical competence and there is a precautionary stance in acknowledging the benefits presented by self-evaluation for promoting further development. This article stands therefore to promote self-evaluation and ignite discourses regarding ethical competency amongst nurses, as there is limited information on self-reporting of students regarding their own ethical competence.

The aim of this article is to describe the analysis of self-reflective narratives of students in handling real-life ethical issues. This is achieved by identifying the key dimensions of ethical competence within the narratives and contrasting these with the ethical framework (principlism and code of ethics), as an ethics instruction received.

## Research designs and methods

A grounded theory qualitative design was used to conduct the study. Open coding, as the first phase of grounded theory, was used to analyse the reflective narratives of midwifery students regarding their own description of ethical competence. This design was suitable for the study because open coding enables line-by-line examination of the concepts and conceptualisation of the underlying patterns of the identified idea (Streubert & Carpenter 2011).

## Participants

Starting in 2013, the institution decided to pilot the use of a portfolio of evidence, and 90 students who were enrolled for midwifery in a 4-year programme in 2013 and 2014 were recruited to participate in the study, by way of submitting their portfolios of evidence. The portfolio of evidence was being piloted using the eight essential competencies for basic midwifery practice as described by the International Confederation of Midwives (2010). One of the basic components of first competencies indicates that a midwife should understand the legal and regulatory framework governing reproductive health for women of all ages, including laws, policies, protocols and the professional guidelines of the International Confederation of Midwives (2010). For the purpose of describing their evidence regarding ethical competence, students were requested to keep a journal of their experiences during their placement in maternity wards with the following instructions:

- Write a reflection report of about two pages and narrate a critical incident in which you think you were ethically competent in your provision of care.
- Identify the skills you employed while dealing with this incident and make note of any person, course or module that assisted you in the accomplishment of this competence.

Out of 90 students, only 60 handed in their portfolios at the end of the year, after 6 months of placement in maternity units. The submission of this portfolio was a voluntary exercise; students were informed that it would form part of a research study and that, by submitting a portfolio, they were consenting to participation. The institution also gave permission for the students to participate. The study obtained an ethical clearance from the Human Research Ethics Committee (Medical) of the University of the Witwatersrand (M150773).

## The sample

The data set consists of the reflection report narratives written by students regarding their competency in handling ethical issues. Students were asked to collect narratives of their own ethical efficiency in handling ethical issues as a portfolio of evidence while placed in different midwifery clinical areas. Fifteen portfolios were selected purposefully, and 14 portfolios that were included formed the sample size. Narratives within the portfolios were considered if they were written in English and showed evidence of completeness. The students were expected to provide evidence of all eight essential competencies for basic midwifery practice and to use the English language for narrating their experiences as a policy of the institution.

## The trustworthiness of the study

The following criteria are recommended for ensuring trustworthiness in qualitative research: credibility, transferability, dependability and confirmability (Polit & Beck 2012). In ensuring confirmability, dependability and credibility the researcher made use of the co-coder to increase the reliability of the findings. The use of a co-coder guarantees that the data compare accurately with the research question, and this also increases the credibility of the results. After the co-coder and researcher analysed the narratives independently, a meeting was set up to discuss and reach consensus regarding the identified themes and categories. Transferability was ensured by the in-depth description of the methodology and metadata. In-depth description of methodology was to enable duplication of the study and contextualisation of data by other researchers, as advised by Brink, Van der Walt and Van Rensburg (2012).

## Data analysis

The analysis of data followed open coding, as described by Streubert and Carpenter (2011), which entails line-by-line examination of the concepts and conceptualisation of the underlying patterns of the identified idea. The codes that were used in this study were based on the definition proposed by Jormsri et al. (2005), which identifies ethical competence as the ability to perceive, act and justify one’s actions based on ethical knowledge, skills and attitudes, as well as the recognition of one’s own feelings and the feelings of others involved in the incident, as described by De Schrijver and Maesschalck (2013). The phrases or concepts from the self-reflective narratives that pertinently portrayed the ability to perceive, act and justify intentions to achieve a good midwifery outcome were used for illustration of the categories.

Then the developed categories of ethical competence were paralleled with codes of ethics and principlism framework, so as to identify suitability or appropriateness. Accordingly, Leget and Borry (2010) affirm that empirical results embody factual normativity and should be tested against normative principles and theories. Therefore, the categories of data were compared against ethical principles enshrined within principlism and codes of ethics.

The categories identified were as follows:

- moral perception
  ■ the ability to identify moral issues
- moral action
  ■ the ability to act
- justification for moral action
  ■ the ability to justify one’s action based on ethical knowledge, skills and attitude
  ■ portrayal of ethical principles and codes of ethics as an account of acquired nursing ethics education.

Fifteen portfolios that were written in English and had evidence of a complete report were selected. Fourteen reflective narratives and reflection reports from the portfolios were analysed, after which data saturation was reached. Permission to use the portfolio was obtained from the head of the institution and consent was obtained from the students the portfolios belonged to. Anonymity of the portfolios was maintained by using numbers instead of the names of the candidates.

### Ethical considerations

The narratives were analysed after ethics approval was obtained from the Human Research Ethics Committee (Medical) of the University of the Witwatersrand (clearance certificate no. M150773).

## Findings and discussion

The themes that were identified when the self-reflective narratives were analysed for ethical competence are presented under the categories that define ethical competence, as described by Jormsri et al. (2005), Kulju et al. (2016) and Meine and Dunn (2013).

## Moral perception

Moral perception is defined as the ability of an individual to recognise and sense that there is a moral issue in a particular situation (Jormsri et al. 2005). Moral perception is considered to be affective in nature, because it involves an experience of emotions, in which one becomes aware of values and their expression in a given situation (Jormsri et al. 2005).

The students were able to recognise that a specific situation presented or had a likelihood of rendering an ethical issue. Moreover, it can be argued that this ability was only based on intuition; Haidt (2001) claims that the initial source of moral judgement is intuition, because it involves a sudden affective value (good–bad, like–dislike) that lacks balanced evidence. This was demonstrated by some messages portraying principles such as avoidance of harm, increasing benefits and autonomy as well as some emotions of students as the moral agents. Examples of responses are therefore given.

### Obligation to beneficence and non-maleficence

According to Beauchamp and Childress (2013) the norms that structure the principles of beneficence include preventing or removing harm, as well as promoting good, while those structuring non-maleficence involve not inflicting harm (Beauchamp & Childress 2013). The participants in this study narrated the principles of non-maleficence and beneficence, and these are described below:

‘The woman that I helped to give birth sustained a vestibular tear that was bleeding. I wanted to suture it, but the sister told me that she need no suturing as she will heal. I did a follow-up on her in postnatal ward. I was worried about the tear.’ (Participant 12)

‘I was told by the head midwife to leave alone a patient who was 9 cm dilated and about to give birth. I was upset with the midwife. The patient nearly gave birth alone.’ (Participant 10)

‘I saw that resuscitation of the preterm baby was not effective, and I thought the mother’s presence was essential.’ (Participant 7)

‘I saw the midwife that was assisting with the birth of a baby with severe prematurity, and she told the mother that the baby is dead, while the baby was still gasping.’ (Participant 4)

Given the emotions of being upset with the midwife and being worried about the tear, one can recognise that the perception of possible harm was also coupled with emotion, as described by Haidt (2001).

It is obvious from the preceding transcripts that the narrators were also moved by their duty to do good, which enabled one of the students to perceive that the woman with vestibular laceration may be harmed by haemorrhage, while ineffective resuscitation of the newborn baby was perceived to result in psychological harm for the mother.

### Obligation to support autonomous decision

Some of the transcripts illustrated the students’ desire to respect the autonomy of their clients. There was an incident in which a student believed that disclosure of all relevant information would capacitate the patient’s autonomy.

‘I met the mother who did not attend clinic because she believed that the iron tablets that were given to treat anaemia with her previous pregnancy had caused her miscarriage. I gave her information regarding the medication and importance of antenatal care, as well as possible causes of miscarriages. I knew ethically I have to respect her choices, I understood her reasoning but, I wanted her to be more knowledgeable regarding the complications.’ (Participant 1)

‘As I was conversing with the husband of one of the mothers, I learnt that he had never witnessed the birth of his children, because he didn’t know how to go about it as [*it*] is not a normal practice in his culture.’ (Participant 6)

‘The mother with eminent eclampsia was reluctant to sign an informed consent for the termination of pregnancy, [*and*] she asked the doctor whether she could have a second opinion [*from*] another doctor, despite the fact that her life was in serious danger and that termination was meant to save her life, instead of the foetus.’ (Participant 8)

These transcripts show that the ability to perceive the situation as being ethically charged was based on the moral judgement grounded within the principles of non-maleficence, beneficence and respect for persons. This was an obligation to do well as stipulated in the code of ethics for nursing practitioners, as well as the principlism framework.

## Moral action

Moral action is a courageous act that is self-defined as being right by the student, as demonstrated in the following narratives.

### Obligation to beneficence and non-maleficence

Moral actions relative to the preceding principles were described within the context in which they happened, as highlighted in the following:

‘Three hours later while the mother was already in postnatal ward, I went to assess and see how she was doing. I found the woman soaked in blood and I took her back to labour ward to be sutured.’ (Participant 12)

‘I went and I was back in [*a*] few minutes because I asked the patient’s husband to come to call me back. The patient nearly gave birth alone.’ (Participant 10)

‘I went to ask the doctor if there [*were*] no other possible solutions in situations like this. After a few hours the baby was taken to high care for palliative care as it was said he was too premature to survive.’ (Participant 4)

‘I asked the Doctor … [*i*]f I [*could*] go and call the mother, as her preterm baby’s condition was deteriorating despite the resuscitation. After [*the*] death of the baby I asked if she would like some privacy with the baby. She agreed and I placed the baby on her chest (skin to skin). I closed the curtains and allowed them to be on their own. After a while I came back and I used the mother’s cues to offer support. I wanted her to have a role in the situation.’ (Participant 7)

‘A patient with obvious uterine contractions walked in, and we were trying to communicate with her, but she couldn’t speak either English or Afrikaans. I used a little bit of Sesotho I learned while in primary school, but, I also requested assistance from one of my colleagues, though we were not best friends.’ (Participant 9)

### Obligation to support autonomy

Some of the narratives clearly demonstrated that the students respected the autonomous position of their patients. The moral actions specific to respect for persons were described as follows:

‘We provided her with all the information and allowed her to ask questions. We phoned her husband and family to ask them to come and support her. I understood her reasoning, but I wanted her to be more knowledgeable regarding the complications. We wanted the family to assist with decision-making.’ (Participant 8)

‘I gave her information regarding the medication and importance of antenatal care, as well as possible causes of miscarriages. I knew ethically I have to respect her choices, I understood her reasoning, but, I wanted her to be more knowledgeable regarding the complications.’ (Participant 1)

‘I made arrangements for the father who wanted to support his wife and witness the birth of his children.’ (Participant 6)

The transcripts indicate that the students were respecting the self-determination of the patients and their clients. They capacitated the patient to be autonomous by providing information, where they felt that lack of information was likely to infringe on self-determination.

## Justification for moral action

Moral justification is concerned with how the students substantiate the moral assertions of their particular act or practice, as to determine whether those practices are good or bad, permissible or impermissible (Scanlon 2013:601). Haidt (2001) argues that the primary or initial source of moral judgement is intuition, as it involves a sudden affective value (good or bad, like or dislike) that lacks balanced evidence or diverse, thoroughly contemplated strategies. Saunders (2009) claims intuitive judgement is motivated by feelings, response to the internal representation of norms and values that are always laden with emotions.

The judgements were made explicit in this study as discussed in the following.

## Justification of autonomous decision

In justifying the autonomous decision of the patients, excerpts such as the following were included. These also illustrate that the students were not reasoning but conforming to a code of ethics and principlism as a moral outlook that was taught in the ethics module:

‘I understood her reasoning, but I wanted her to be more knowledgeable regarding the complications. We wanted the family to assist with decision-making.’ (Participant 8)

‘I believed that a patient should be treated holistically and be informed.’ (Participant 3)

‘The Asian woman refused the support of the husband as well as to eat specific food while in labour because of her culture. I knew ethically I have to respect her choices.’ (Participant 5)

‘I felt that [*i*]t was the father’s right and he was not aware of it.’ (Participant 6)

The narratives indeed reveal that the midwifery students considered and also applied the normative precepts that were relevant to the situation. This is what Beauchamp and Childress (2013) call a top-down model that relies on the application of precepts in an obligatory way rather than determining what could have been permitted or prohibited. This demonstrates that students reflected on the codes of ethics and principlism while justifying their moral action. Some of the excerpts illustrate that there were cases where autonomous decision-making was deemed to be limited and the narrators felt obliged to facilitate it. Respect for autonomy is concerned with honouring the rights of the patients to self-determination or informed decision (Dhai & McQuoid-Mason 2011). To be autonomous, the theory asserts that the individual must be free from coercive powers and must have the capacity for intentional actions that are free from conflicting wants and desires (Beauchamp & Childress 2013). Thus, one would be rendered non-autonomous if one has not reflected on his or her wants or desires (Beauchamp & Childress 2013), and these were not made evident within the reflection reports.

## Justification for beneficence and non-maleficence

Some of the assertions that were used to justify the beneficence action include the following:

‘It was obvious that the language was a barrier to good care for the woman.’ (Participant 9)

‘I felt that it [*was*] not right.’ (Participant 13)

The narratives entailing the acts of beneficence lacked dialogue with the patient and therefore seemed to provide one-sided information. Quotes such as ‘I felt that it [*was*] not right’ or ‘I was being empathetic’ can be seen in the light of intuition response emanating from the internal representation of norms and values. This concurs with Haidt’s (2001) claims that such judgements are laden with emotions and have sudden affective valance with no balanced evidence or thorough, searched-for diverse strategies with regard to the situation. Although the narrators gave an explanation for their moral action, their sources of knowledge were limited in nature. The principles that were used to justify ethical actions were the respect for persons and beneficence.

The student could have properly reasoned his or her actions of advocating for the mother or capacitating the knowledge of the client if the mother’s opinion regarding her needs and desires had been stated. However, this was not the case in these situations. The preceding excerpts show that beneficence and respect for persons were the only principles that were used to justify moral actions. These narratives do not display a set of principles that were involved and therefore lack diversified strategies to bring about a logical conclusion to the argument. Campbell (2014) indicates that a justified decision must have evidence or facts from the event as well as diversified strategies for bringing about coherence and soundness to the decision-making, especially in situations of competing values and norms. Although some of the quotes reveal that there were competing norms, one would expect the use of diverse strategies. This claim is demonstrated by the following:

‘The Asian woman refused the support of the husband as well as to eat specific food while in labour because of her culture. I knew ethically I [*had*] to respect her choices.’ (Participant 5)

‘[*A*] mother with imminent eclampsia was reluctant to sign an informed consent for termination of pregnancy. I understood her reasoning, but I wanted her to be more knowledgeable regarding the complications. We wanted the family to assist with decision-making.’ (Participant 8)

Excerpts like these call for dialogue, as there are competing norms between the patient and healthcare provision. These excerpts depict compliance to principles as rules or obligations. Conforming to these principles is, as Meine and Dunn (2013) clarify, too simplistic to assert that there is ethical competence, if what is necessitated is the application of principles, laws and formal rules. Instead, competence should also entail communication and argumentation skills, as well as confidence and emotional intelligence, in dealing with views that are different from one’s own (Meine & Dunn 2013). As described by Beauchamp and Childress (2013), a post hoc judgement and final analysis are needed where one practises reflective equilibrium, in which one is expected to critically analyse his or her behaviours against a whole range of moral knowledge, skills and attitudes so as to support his or her moral assertion. In such cases Lechasseur et al. (2016) suggest that there is a need to employ the process of reflective equilibrium, which will assist in harmonising the total set of principles and the empirical claims involved. Jormsri et al. (2005) further indicate that moral reasoning must not merely be defined by mastering the principles found in the codes of ethics, but there must be a capability of analysing the situation and reflecting on the feelings, intuition and experiences of others.

## Logical justification

Logical justification of a moral action requires moral discourse that must be based on logical reasoning and critical thinking as well as appraisal of values by all the stakeholders. As described by Lechasseur et al. (2016), such a moral justification extends beyond the individual’s normative knowledge, to include the beliefs and values that are put forward by the other stakeholders within the context of the situation.

Only two narratives met this requirement, as demonstrated by the following excerpts:

‘I saw that resuscitation of the preterm baby was not effective, and I thought the mother’s presence was essential. I asked the doctor if I [*could*] go and call the mother, as her preterm baby’s condition was deteriorating despite the resuscitation. I wanted her to have a role in the situation. I was empathetic and felt I should provide hope to the hopeless.’ (Participant 6)

‘The mother was told that the baby [*was*] dead, while the baby was still gasping, and I asked the doctor if there [*were*] no other possible solutions in situations like this, as it was said he [*was*] too premature to survive. I was advocating for the mother.’ (Participant 4)

The basis of the extracts above falls within Gallagher’s (2006) notion of ethical competence, which stipulates that nurses need to demonstrate critical reflection about what they know, who they are and what they do, in order to bring about an ethical practice. Even though the preceding decisions made can be viewed as translating from moral intuition and emotions, as suggested by Haidt (2001), the narrator considered the context and the other stakeholders before calling the mother when the child’s condition was deteriorating. Furthermore, the narrator asked the doctor whether there were no other alternatives of care for newborns facing death. This reveals that the discussion with a doctor was asking for consensus to be reached before calling the parent. It is clear that there was involvement of emotion and intuition coming from experience and obligation to do well, while at the same time the feelings of others were envisaged.

## Discussion

The students’ narratives regarding their own ethical competence were analysed to check whether there was evidence of such a skill. The findings revealed that the students were able to perceive moral issues and to engage to some extent with certain values espoused in a specific situation. It is obvious that students were able to perceive that certain situations carry either a morally sound or morally wrong practice. The transcripts on moral perception concur with Jormsri et al. (2005), who state that commitment to professional values is necessary for the awareness of moral issues. In this study, the students were committed to the principles of beneficence and non-maleficence. In addition to professional values, empathy or ‘being in the shoes’ of the mothers compelled the student to see that certain practices during birth were considered essential or harmful. One aspect stated by Jormsri et al. (2005) is the involvement of the affective and cognitive domains in moral perception. This aspect was also evident in the narratives of the students, as their emotions were charged whenever they suspected potential harm to the patients. The use of the affective domain in moral perception is affirmed by Wisnewski (2015), who argues that patients’ distress and anxiety are a fundamental embodiment that induces sympathetic moral perception.

Despite the fact that students did not mention principlism per se as normative grounding for their moral actions, their acts testified to some of the four principles. The principles that were emphasised were respect for autonomous choice, beneficence and non-maleficence. A limited number of the narratives showed that the students were not acting morally from habit but that they considered different knowledge, such as human rights and midwifery practice. It can therefore be concluded that the application of principlism and a code of ethics was limited to the use of beneficence, non-maleficence, respect for autonomy, as well as advocacy. Although moral perception and moral actions were demonstrated, there were no epistemic reasons given as to why they felt that their assertions should be believed as having moral worth. The assertions from the narrative were not in accordance with the preferred moral justification by Beauchamp and Childress (2013) of reflective equilibrium process. In reflective equilibrium, all the principles applicable to a situation are to be theoretically in harmony, so as to maximise the coherence of the overall set of beliefs. Instead, the findings reveal that an empathetic moral intuition that was aimed at doing good and avoiding harm was evident in most narratives. Saunders (2009) describes moral intuition as an easy and immediate judgement that is made without any conscious effort. Taking empathy into account, as the trait was explicitly portrayed, it may be said that the caring nature of nursing prompted the students to use moral intuition as identified in this study. It must, however, be understood that, although valuable, moral intuition is unreliable and subject to all kinds of bias (Brun 2014). Thus, for a judgement to be morally reasoned, a conscious effort of reason, harmonisation of the total set of principles and the empirical claims involved are to be reasonably reconciled (Lechasseur et al. 2016).

## Conclusion

The objective of the study was reached as analysis of the self-reporting ethical competence revealed that there was moral perception and moral acts. Even though the students considered themselves to be ethically competent in their midwifery care in handling ethical issues, this competence was found to be limited because the self-reflective narratives did not demonstrate all three dimensions of ethical competence, namely moral perception, moral judgment and moral behaviour. Moral reasoning was found to be limited to intuitive moral reasoning during the analysis of the narratives. Intuitive moral justification is to be followed by a reasoning that is critical. This was not the case in this study, as the majority of students did not support their assertions logically. The narratives revealed that there was no line of reasoning; instead, the students were conforming to the ethical principles, codes of ethics and personal values of their choice. A follow-up study on the professional life of these students would be valuable as analysis of the impact of self-evaluation on their process of ethical competence.

Given that the analysis of the self-reporting ethical competence demonstrates partial ethical competence amongst the midwifery students, it is recommended that there should be discussions in institutions of nursing education regarding moral theories in general, especially institutions that have for years relied solely on the four ethical principles and the codes of ethics for the acquisition of ethical competence. In particular, the discussions should be aimed at examining whether it is necessary to immerse students in diverse moral theories such as virtue ethics, care ethics, deontology and utilitarianism, and run the risk of limited knowledge, or to select and study in depth the moral theories that are essentially pertinent to nursing and midwifery care. If the need is to concur with the current curriculum for nursing ethics education, it is recommended that the ethical principles as stipulated by the proponents of principlism be discussed fully and not be conveyed to students as abstract concepts. A model such as reflective equilibrium should be taught, so as to help students to reconcile all ethical principles with their norms and values for the purpose of coherent decision-making pertinent to patient-centred care.

## Limitations and further research

This was a small-scale study conducted at one institution with a limited number of participants, and as such it cannot be generalised to the entire population of student midwives and nurses in South Africa. Furthermore, it was a retrospective study in which a follow-up with the participants for further explanation and clarification of certain issues mentioned in the narratives was not possible. It is therefore essential that another study be conducted in which both quantitative and qualitative data may be generated from all the institutions of nursing education in South Africa. Such a comprehensive study would provide a bigger picture of the state of nursing and midwifery practice regarding training for ethical competence.
